# Music festival drug checking: evaluation of an Australian pilot program

**DOI:** 10.1186/s12954-022-00708-3

**Published:** 2022-11-19

**Authors:** Anna Olsen, Gabriel Wong, David McDonald

**Affiliations:** 1 Medical School, The Australian National University, Florey Building 54 Mills Road, Canberra, ACT 2601 Australia; 2 Centre for Social Research Methods, The Australian National University, Level 2, Beryl Rawson Building, Canberra, ACT 2601 Australia; 3 National Centre for Epidemiology and Population Health, The Australian National University, Mills Road, Canberra, ACT 2601 Australia

**Keywords:** Drug checking, Pill testing, Festivals, Evaluation, Australia

## Abstract

**Background:**

This paper explores the feasibility of delivering a music festival-based drug checking service in Australia, evaluating service design and stakeholder acceptability.

**Methods:**

Questionnaire and interview data were collected from adult service users and key stakeholders. A mixed methods approach was used to analyse the data on implementation, impact and acceptability.

**Results:**

The trial service tested 170 substances with more than 230 patrons (including individuals who attended in groups). Adult service users had an average age of 21 years. Voluntary participation in the evaluation resulted in 158 participants completing the pre-service questionnaire, most of whom also completed the post-service (147 participants). Eleven in-depth qualitative interviews were conducted with patrons in the weeks following the drug checking. Concordance between what the patron expected the drug to be and drug checking results occurred in 88 per cent (*n* = 139) of the sample. Evaluation results show that the experience of testing and the accompanying harm reduction brief interventions positively impacted on patrons’ self-reported drug harm reduction knowledge, trust of health providers and stated drug use intentions. The service was received positively by service users.

**Conclusion:**

This is the first independent evaluation of a pilot drug checking service in Australia. Consideration of operational feasibility and self-reported behavioural change suggests that the program was successful, although communication about the interpretation of drug checking results could be improved. Future studies should develop strategies for follow-up and consider the applicability of behavioural change theory.

## Introduction

Drug checking or pill testing services have existed for over 50 years and now operate in more than 20 countries [1]. Despite this long history, little research assessing feasibility or impact has been published in peer-reviewed journals. As more countries trial drug checking services, the number of peer-reviewed evaluations have grown [2–7]. This paper describes the evaluation of the first government-sanctioned drug checking service in Australia with the aim of assessing feasibility and comparing findings with the growing international evidence-base.

Drug checking services (also known as pill testing) are a health intervention aimed at performing chemical analyses to assess the contents of illicit substances, generally also accompanied by health information about reducing the potential harms associated with drug use [8]. There are a range of service models providing different levels of access to the general public. Aggregating data from drug samples, drug checking services can also provide insight into trends in the unregulated drug supply [2]. While service models include fixed site, mail-in services and others, the debate around provision of drug checking services in Australia has largely focussed on face-to-face services at music festivals [9].

Music festivals are a common location for Australians to engage in illicit drug use [10, 11]. Australian research shows that people who use illicit drugs at festivals perceive risks associated with their drug use [12] and want information about the contents of the substances they intend to use [11, 13–17]. Research suggests that festival-goers and other young people who take drugs currently attempt to find out information about their drugs through unreliable sources such as friends who have used the drug previously, dealers and websites [17].

Research on acceptability of drug checking shows a high interest in using testing services at festivals and elsewhere. In the Australian context, a questionnaire of general festival-goers found that half (54%) indicated that they would be highly likely and a third (33%) somewhat likely to use free drug checking services in a festival setting [17]. Most (86%) believed that drug checking services could help users to reduce harms associated with drug use. Another questionnaire with Australians who use psychostimulants found that 94% reported that they would use a festival-based drug checking service [15]. However, these data tend to rely on patrons commenting on hypothetical services and little is known about the perceived quality, value and usefulness of drug checking among those who have attended a service. In Australia, only the Australian Capital Territory has permitted the trial of a drug checking service, carried out in 2018 and 2019. The service was independently evaluated in 2019 [18], and is the subject of this paper.

One of the areas of criticism in the debates around drug checking services and other harm reduction initiatives is the lack of evidence around ‘good practice’ [19]. This refers to a lack of implementation standards but also lack of implementation evidence. One of the areas of knowledge generation essential for evaluation and implementation research is the experience of and outcomes valued by stakeholders [20]. Incorporating experience and context into healthcare assessment provides evidence on how patients make sense of their health, whether the health information provided is considered appropriate and useful as well as the perceptions of those involved in the development and delivery of the service [21]. This involves asking questions about relevance, applicability and usefulness in order to provide information about whether an intervention should proceed and if so, how.

Given the need for further evidence of the feasibility of providing drug checking in the Australia context, an external, independent evaluation of the 2019 ACT drug checking trial was conducted. The overall goal of this evaluation was to evaluate the implementation, impact and acceptability of the trial drug checking service operating out of a festival in Australia’s capital city. To date, few independent evaluations of drug checking services are available and none in the Australian context. Given the limited independent research conducted on drug checking services, these data provide insight into the value and usefulness of the service, as well as feasibility of potential future services.

### The ACT drug checking trial

The first government-approved drug checking trial to be implemented in Australia was conducted in 2018 at the Groovin the Moo (GTM) festival in Canberra by Pill Testing Australia (PTA) [22]. A second trial [15, 21] (the subject of this evaluation) was approved to run at the GTM festival in Canberra a year later (in 2019). GTM is a one day, annual pop-music/dance festival, held in diverse venues across Australia and attended by over 23,000 in Canberra in 2019. The 2019 drug checking trial was supported by Australian Capital Territory (ACT) Health and ACT Policing with agreements established to allow the service to provide information to patrons without interference. The service was designed, implemented and financed by Pill Testing Australia.

The front-of-house, onsite drug checking model was informed by a harm reduction approach that seeks to advise patrons about the contents of the substances they are considering taking and deliver harm reduction information, while also providing important data on the drugs in circulation to health and law enforcement agencies. Drug checking was undertaken by trained chemists using the Fourier-transform infrared spectroscopy (FTIR) approach; two ALPHA II machines were used [21].

The drug checking service was co-located with medical services, set up in an existing shed at the festival grounds. There was a divider and separate entrance between the two services. Patrons entering the service were assessed for eligibility and asked to sign a waiver form that absolved the service providers from liability for any adverse events, before submitting a scraping of the substance for testing. Not all service users presented a drug for testing as some entered the service in a group with only one drug sample, accompanying their friend whose drug was to be tested. It is unknown how many of the group members planned to use drugs at the festival. After the substance was tested, chemists and medical staff provided patrons with the result and reiterated that no level of drug use is ‘safe’. Patrons then received a brief personalised harm reduction intervention from a DanceWize peer educator to discuss the risks involved in consuming the substance and how to minimise these risks. The chemists advised patrons of what drugs they found in the samples proffered, the medical officers responded to any questions about the medical risks involved in using the drug, and the peer educators focused on harm reduction. The service was explicitly non-judgemental, neither promoting nor condemning drug use. Referrals to health or alcohol and drug services were provided where necessary, primarily as an outcome of patron engagements with the peer educators. A card with their sample number was provided to patrons to be given to emergency services in the event of a drug-related presentation to allow emergency services to identify the substance taken through the drug checking service.

## Methods

The evaluation used data from pre- and post-service questionnaires and follow-up interviews with patrons. On entering the service, patrons were screened by the evaluation team for eligibility for participation (including being aged 18 years or older, and not intoxicated. Patrons under 18 years of age were excluded from the evaluation (but not from the drug checking service) by the provisions of the Australian National University’s (ANU) ethical approval of the evaluation. Those identified as eligible were invited to complete a questionnaire before presenting their substances for testing or accompanying a friend who was presenting a substance for testing. A total of 234 patrons entered the service. Of these, 22 declined to enrol in the evaluation and 53 were under the age of 18 years of age (and hence were excluded from the evaluation), resulting in 159 people participating in the evaluation. One of these was subsequently excluded from the analysis as they knowingly presented a sample of candy for testing, leaving a total of 158 valid evaluation patrons. Once they had received their testing results and completed the brief intervention delivered by the trained peer educators, evaluation patrons were invited to complete a second questionnaire. 147 of the 158 pre-test patrons (93%) completed the post-service questionnaire. The pre- and post-service questionnaires were self-completed on paper by the patrons, under the oversight of trained evaluation personnel from the ANU. A unique ID was given to each evaluation participant. This ID was written on both the pre- and the post-service questionnaires allowing individuals’ data to be linked.

Thirty patrons agreed to be re-contacted at a later date. Four months after the festival, eleven patrons were able to be re-contacted and agreed to the in-depth semi-structured interview. The topics covered included basic demographics, patrons’ accessing the service, their expectations about their drugs prior to testing, their attitudes and drug-related behaviours prior to the festival, their experiences of the service, their attitudes and behaviour soon after they left the service and in the following months. These follow-up data provide novel information about patron behaviour.

### Analyses

Descriptive statistics were used to capture patrons’: (i) demographics; (ii) expectation of the drug content and resulting concordance between the expected and actual results; (iii) patrons’ intended consumption of drugs; and (iv) attitudes to the service. In addition, analyses were conducted to examine changes regarding patrons’ harm reduction knowledge and patrons’ trusted sources of drug information before and after patrons receiving drug checking and brief intervention services. Patrons’ harm reduction knowledge was measured using a 5-point Likert scale (from 1 equals ‘very poor’ to 5 equals ‘very good’) before and after drug checking. Hence, the Wilcoxon signed-rank test was employed to determine whether there is a difference in mean ranks of harm reduction knowledge before and after drug checking. The difference in mean ranks will reveal the impact of drug checking on self-perceived harm reduction knowledge. Patrons’ trust towards 10 sources of drug information (e.g. peers, dealers, Internet) was measured separately yielding ‘yes/no’ dichotomous responses for each source of drug information before and after drug checking. The McNemar test was employed to determine whether differences exist on patrons’ trust towards each source of drug information. Differences on patrons’ response will reveal the impact of drug checking on patrons’ trust towards alternative sources of drug information.

Interviews were digitally recorded and transcribed verbatim. Thematic analysis was used to identify and analyse themes within the data [23]. Transcripts were re-read and re-coded, systematically comparing interviews for themes related to the evaluation questions and topics from the research literature. All identifying information, aside from profession, gender and age, has been removed.

## Findings and discussion

### Questionnaire patrons

Slightly fewer than half of the evaluation patrons self-identified as female (*n* = 76, 48%), slightly over half of the patrons self-identified as male (*n* = 81, 51%), and one participant self-identified as another gender (1%). The age range was 18–51 years old; the average (mean) age was 21.18 years (SD = 4.59). Most of the patrons (*n* = 139, 88%) reported prior experience of consumption of an illegal drug other than cannabis.

Among the patrons of the evaluation, 106 patrons responding to the pre-service questionnaire (67%) were there to present a drug for testing while the remaining patrons were there to accompany other patron(s). According to the post-service results, most of the patrons (141 out of 147 patrons, 96%) personally received the test result from staff or were present when the result was given. Most of the patrons (123 out of 147 patrons, 84%) who provided post-test questionnaire data also received the brief intervention.

### Interview patrons

Of the eleven service user patrons who participated in a follow-up interview, five were female and six were male. Patrons were aged between 19 and 29. Six patrons resided in NSW and five in the ACT. Ten of the eleven had used an illegal drug other than cannabis before the festival, and only three had spoken to a healthcare provider about drug use before. Most patrons presented a single substance for testing, though two presented two samples. Three patrons presented substances they had found on the ground at the festival.

### Testing results

A total of 234 patrons entered the service, twice the number of patrons who used the service in 2018 [22]. The testing service operated between 11:00am and 9:30 pm on the day of the event. According to service data, most patrons entered the service between 1:00 pm and 6:00 pm, during which time 126 samples were analysed at an average rate of one sample every 2–3 min. This rate of testing was close to capacity for two instruments staffed by four qualified chemists.

In the pre-test questionnaire, participants were asked ‘What do you think the drug being tested is?’ As shown in Table 1, most (*n* = 136, 89%) believed that the drug was MDMA, with just four expecting MDMA mixed with methamphetamine, two cocaine and one ketamine. In nine cases, the participants did not know what the substance was or the response was unclear.

**Table 1:** Participant expectations of drug type and sources of drugs

	Number	Per cent
*Expectation of drug type (n* = *152)*		
MDMA	136	89.5
Cocaine	2	1.3
Ketamine	1	.7
Mixed MDMA and methamphetamine	4	2.6
Unidentified	9	6
*Reasons for the expectation (n* = *155)*		
*‘Already tried it’*		
Yes	56	36.1
No	99	63.9
*‘That is what I was told by the person supplying the drug’*		
Yes	102	65.8
No	53	34.2
***‘****I have tested it using a home drug testing kit’*		
Yes	3	1.9
No	152	98.1
*Other*		
Yes	13	8.4
No	142	91.6
*Source of the tested drug (n* = *156)*		
Dealer (face-to-face)	29	18.6
Friend	78	50.0
Relative	1	.6
Acquaintance	3	1.9
Online	3	1.9
Don’t know	11	7.1
Rather not answer	5	3.2
Other	6	3.8
Dealer and friend	18	11.5
Friend and relative	2	1.3
*Location of drug purchase (n* = *155)*		
Inside the venue	10	6.5
Outside the venue	120	77.4
Don’t know	19	12.3
Rather not answer	6	3.9

When asked, ‘What makes you think that?’, the majority (*n* = 102, 66%) stated that was what they had been told by the person who supplied the drug. Fifty-six participants (36%) had already tried the drug, and just three had tested it using a home testing kit.

Most participants reported that the drug was purchased outside of the festival venue (*n* = 120, 77%), with just ten reporting acquiring it inside the venue, nineteen reporting ‘don’t know’, and six stating that they would ‘rather not answer’. A range of sources of the drugs presented for testing were reported. The largest source was friends (50%), followed by dealer (19%) and dealer and friend (11%), with far smaller proportions reporting acquaintance, online, both friend and relative, and relative. Eleven participants (7%) stated that they did not know where the drug came from.

According to the service data recorded by PTA, MDMA was the predominant substance identified, and to a much lesser extent cocaine, ketamine and methamphetamine [24]. The cathinone drug *N*-ethyl pentylone was tentatively identified in two samples provided by festival medical personnel and five samples presented by patrons. This drug has been associated with deaths and mass casualty events in the USA and New Zealand [25, 26].

### Concordance between drug expected and drug tested

Patrons’ expectations of the contents of their substance and what was found through testing were compared. It is important to note that we are reporting on what the patrons understood to be the substances found through the chemical analyses, based on what they recalled being advised by the chemists and the peer counsellors. Their perceptions and recollections are not necessarily 100% correct. Overall, most (88%) of the patrons had a generally accurate perception of the contents. Slightly more than one-tenth of patrons (*n* = 17, 12%) had drugs confirmed to be different from their expectations. All of these 17 patrons found the lack of concordance to be ‘somewhat’ or ‘very’ surprising. Approximately half of the patrons who reported concordance between their expectation and the actual content of tested drugs also reported being ‘somewhat’ or ‘very’ surprised. The link between concordance of drug content and patrons’ intended behaviours (e.g. consumption or discarding tested drugs) is further discussed below.

### Impact on patrons’ harm reduction knowledge

**Fig. 1:**
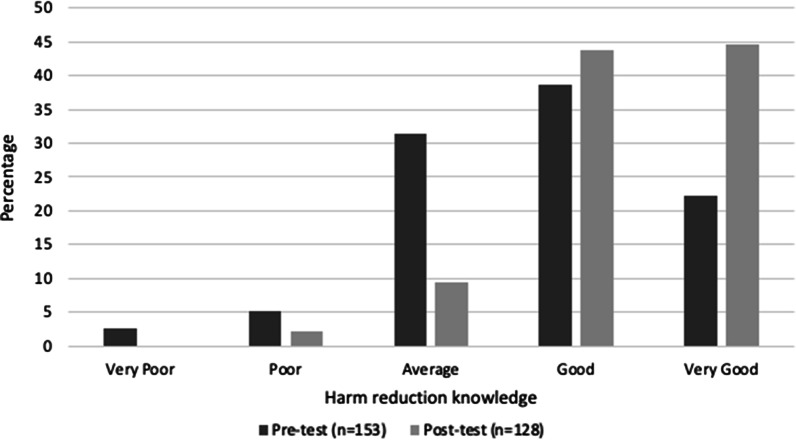
Self-reported knowledge on harm reduction before and after the service

One of the intended primary outcomes of the testing service was to improve patrons’ knowledge of how to prevent potential harms associated with drug consumption (especially consumption of the type of drug that had been tested). Self-reported knowledge was measured from ‘very poor’ [1] to ‘very good’ [5]. Results indicate that a higher proportion of patrons reported having ‘good’ (44%) or ‘very good’ knowledge (44%) of harm reduction post-service when compared to pre-service (with 38% of ‘good’ and 23% of ‘very good’ knowledge) (see Fig. 1). Such a change in perceived knowledge of how to prevent the potential harms is reflected by the Wilcoxon signed-rank test (*z* =  − 6.202, *p* < 0.000) between pre-service (*M* = 3.73, SD = 0.97) and post-service (*M* = 4.30, SD = 0.74) data. The effect size suggests that the drug checking service had a medium to strong impact on enhancing participant’s perceived knowledge (Hedges’ *g* = 0.659).

### Impact on patrons’ intended consumption of the tested drugs, and of other drugs, at the festival

One indication of behavioural change is disposal of substances after receiving information. Service data show that upon learning about the potential harms of *N*-ethyl pentylone, all seven patrons in possession of a drug containing that substance discarded the drugs [24]. According to our evaluation data, nine patrons reported in the post-testing questionnaire that they intended to discard their drugs in the amnesty bin and two reported they would discard their drugs somewhere else. That is, excluding those who reported having no drug to discard (12 out of 141 valid responses), slightly less than one-tenth (8.6%) of respondents reported that they would discard the drugs they had tested. In addition, 16 respondents (12.4%) were unsure whether they would discard their drugs.

Another indication of behavioural change assessed in the evaluation was change in patrons’ reported intention to consume drugs after attending the drug checking service. More than 40 per cent of patrons who used the service reported that they intended to reduce the quantity of drugs or not use them at all. The majority of patrons reported that they were not going to use more drugs during the festival than they had intended prior to accessing the service. Many respondents reported intention to adopt less risky drug consumption on the day. This included using no drugs (7%), only alcohol (i.e. not consuming the tested drug) (6%), or less drug (i.e. not consuming the tested drug as intended) (28%). Only 19% (*n* = 26) stated that they planned to use the same amount as they intended prior to testing, and just 8% (*n* = 11) stated that they would use more of the drug than originally planned. Notably, one fourth of the patrons (26%) were not sure about their drug using intentions.

The follow-up qualitative interviews with patrons provide useful information regarding intention to use and actual behaviours on the day, contextualising the above questionnaire data. Many interviewees reported that the quantity of drugs that they intended to use did not change after testing, as the drug was identified to be what they expected. However, patrons reported behaviour change resulting from their use of the service which was not captured by the questionnaires. Interview data suggest that members of this group were looking for confirmation of the contents of the presented drug, and information about how to reduce potential harms. Many interview patrons indicated that their intention to engage in harm reduction behaviours did increase. These reported behaviours included not taking all of the substance/s at one time, increasing the amount of time between consumption of substances, and being aware of overexertion and hydration in order reduce the potential harms of these drugs. This finding suggests a more informed drug consumption post-service and is well supported by the aforementioned significant self-reported improvement in patrons’ knowledge relating to harm reduction.

I was still planning to take it, which I did. But I figured I'd have to be less active since in addition to the amphetamine I had earlier that day, I wouldn't want to put too much stress on my heart ... Less time dancing. Male, 21

Yeah, I think I got a water, a few waters, throughout the day because obviously that’s good to do and, yeah, it just made me, I guess, conscious about the fact of what I’m doing and looking out for my friends, and it didn’t have a massive impact because fundamentally the pill, in my opinion, was as safe as it can be. But in terms of all the other things surrounding that, like, checking on your friends, having water, it certainly jogged my memory and made that front of mind. Male, 23

### Impact on patrons’ trusted sources of drug information

**Fig. 2:**
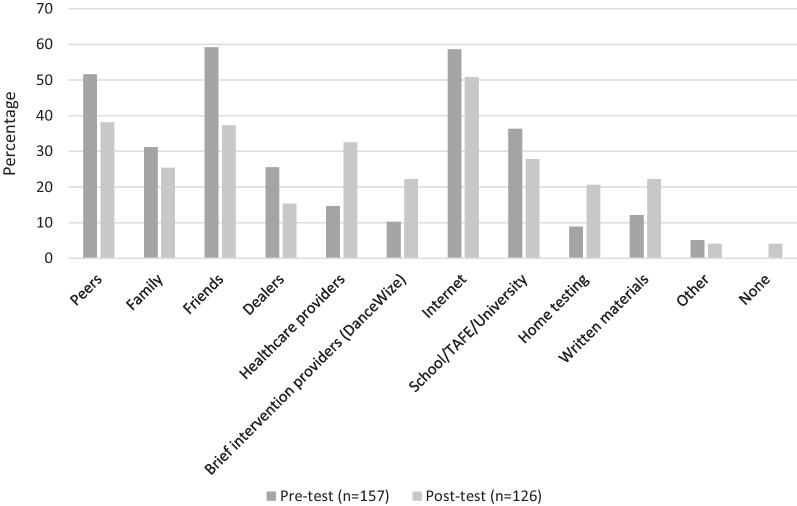
Respondents’ selection of sources of information on drugs, pre- and post-testing

In the pre-service questionnaire, patrons were asked to identify the sources of information that they use to find out about drugs, and the question was repeated in the post-test questionnaire to identify any changes. The post-service questions focused on respondents’ intended use of information sources in the future. Results from a series of McNemar test highlight a dramatic increase in the proportion of respondents who reported that they would, as a result of the drug checking service, be willing to use healthcare providers (up from 14.6 in pre-service to 32.5% in post-service; *p* < 0.001), brief intervention providers/peer counsellors (from 10.2 to 22.2%; *p* < 0.001), home drug checking (from 8.9 to 20.6%; *p* = 0.005) and written materials (from 12.2 to 22.2%; *p* < 0.05) as their sources of information on drugs (see Fig. 2). There was also a tendency for respondents to report that they would be less likely to rely on sources that appeared to be popular during the pre-service data collection. The most obvious declines were observed in the use of information from peers (down from 52 to 38%; *p* = 0.001), friends (from 59 to 37%; *p* < 0.001) and drug dealers (from 25 to 14%; *p* < 0.05). Among all resources, the Internet remained one of the most popular sources of information on drugs with no significant changes in respondents’ selection.

These pre-service/post-service results are echoed in the follow-up interviews, where many patrons talked about how their experience in the drug checking service impacted on their attitudes towards particular sources of information. Overall, people discussed trusting the information from friends and dealers less, and information from health services and institutions more.

… I probably wouldn't trust … my dealer as much, because he had no idea that the pills were that strong … I actually talked to him about it, and he presumed they were good, but he didn't realise they were that pure. So I suppose I wouldn't trust him as much on his recommendations. Male, 21

… the government reports, the ACT, seem more trustworthy now that they have actually field tests to back it up. Male, 21

Just sort of the education that I guess the government gives you. And sort of more trust in sites that sort of preach harm minimisation, because they don't tell you that the drug is good and that you should do it, they just tell you, they realise that people are going to take drugs, no matter what, and this is how to do it safely. So they tell you how to do it safely, while still be telling you about the very real risks of drug use in general. Female, 20

### Approval of the service

The service was received positively by patrons (see Table 2). Most patrons rated the service good or very good (98%), had high confidence in the testing equipment (92%) and said the information they received from both drug checking staff (chemists) and brief interventionists (peer educators) was with good or very good clarity (97%). Ninety-seven per cent of patrons reported that they would use the service again if it was available. Follow-up interview data indicate that patrons value the opportunity to discuss their drug use in a non-judgmental environment and found the information provided to be useful.

It was good, it wasn’t judgmental, it was insightful. Female, 22

… it was a really positive experience. Everyone was really approachable and I guess you kind of forget that when in the media it’s always so negative. And, again, like I said before, being an anxious person, I was worried that there might be judgement behind their words but it was a safe space in there which was really nice. Female, 25

I was also happy with the feedback and the advice I got from the doctors there … I thought it was really well done in efficiently getting in there and the combination of speaking to a doctor and then the counsellor, slash peer supporter, is really good, I think, and it didn’t feel rushed or feel like the people all just read from a script of what she needs to say or he needs to say to you, it was tailored advice relevant to me and delivered in a manner which made me feel comfortable. Male, 23

**Table 2:** Perceptions of service quality

	Number	Per cent
*Overall rating of service (n* = *128)*		
Very poor	0	0
Poor	0	0
Average	2	1.6
Good	14	10.9
Very good	112	87.5
*Confidence with regard to the drug testing equipment (n* = *140)*		
Not at all confident	0	0
Only slightly confident	0	0
Somewhat confident	11	7.9
Fairly confident	36	25.7
Very confident	93	66.4
*Rating of information provided by drug checking staff (n* = *129)*		
Very poor	0	0
Poor	1	.8
Average	1	.8
Good	19	14.7
Very good	108	83.7
*Rating of information provided by brief intervention staff (n* = *128)*		
Very poor	0	0
Poor	0	0
Average	4	3.1
Good	16	12.5
Very good	108	84.4
*Overall clarity of information provided (by both drug checking and brief intervention) (n* = *128)*		
Very poor	1	.8
Poor	0	0
Average	3	2.3
Good	14	10.9
Very good	111	86.0

## Discussion

The paper describes the independent evaluation of Australia’s first government supported drug checking service. As has been shown in international studies of similar services [2, 3, 5, 27], Australian festival-goers were willing to submit substances for analysis and engaged in the harm reduction health advice provided by medical staff and peers.

The service provided valuable new information about the drug market at the time of the festival. The majority of patrons (88%) believed that they were in possession of MDMA and the chemical analysis confirmed that the substance was most likely MDMA. A substance associated with a high risk of overdose and death, the cathinone drug *N*-ethyl pentylone, was found in 7 samples. Service records suggest that all of these samples were disposed of. Overall, slightly less than one-tenth (8.6%) of respondents reported that they would discard the drugs they had tested or were unsure whether (12.4%) they would discard their drugs. This is a lower disposal rate than reported in some other studies [2]. International research shows that low concordance between expected drug and identified drug is associated with high rates of non-use and disposal [28, 29]. Thus, the lower rate of disposal in this study compared with some other research is likely related to the high level of concordance between what patrons expected the drug to be and what the drug was identified as.

A common misunderstanding about drug checking services is that drug disposal and abstinence are the only measure of evidence for behavioural change [30]. As a harm reduction service, the intention is to reduce harms from drugs should people go on to take them after receiving information and/or other resources. In the context of this service, involving primarily young people at a music festival, most patrons presented what they thought was MDMA and analyses confirmed the presence of this drug. The majority of patrons reported that they were not going to use more drugs during the festival than they had intended prior to accessing the service. This is consistent with previous studies with similar research questions where patrons said they would not consume the tested drug as intended and would follow harm reduction advice during consumption [27]. As found in this evaluation and others, non-concordance between patrons’ expectation of what a substance is and what a substance is identified to be commonly leads to reduced intention to take that substance [3, 4, 29, 31]. Conversely, concordance between expectation and identification is associated with stable or increased intention to take a substance.

Considering the rates of disposals and drug taking intentions across published evaluations, it is clear that these behavioural measures are acutely impacted on by the drug market at the time. Behaviours, particularly modification of drug consumption, cannot be measured in isolation from the broader context. That is, the drug market at the time of data collection should also be considered when interpreting patron behaviours around disposal and intended use. Furthermore, the type of festival or other event will impact on the types and proportions of substances used by patrons and brought in for testing [7]. Caution is required comparing measures across evaluations as they reflect a variety of settings.

It is not often well described in the debate or literature about drug checking services that in addition to chemical analyses, health information is generally provided. As described above, in this drug checking service, DanceWize peer educators provided harm reduction brief interventions. Findings from this evaluation, as in others [4, 32], suggest that these health/harm reduction messages have an impact on patrons. There was a significant increase in self-reported capacity to prevent the potential harms of drugs after attending the service. This finding is consistent with existing literature from Europe [33]. Such an increase in self-reported knowledge reflects a success in information provision and dissemination. Although the complex process of translating new knowledge into behavioural change should not be underestimated [34], the provision of preventive and ‘safer use’ messages may reduce adverse consequences (e.g. intoxication and overdosing).

Exploring the integration of drug checking service data with other data sources would provide useful analytical and practical outcomes, in particular interpreting the service utility in the wider context of drug markets, drug trends and health issues. Commonly, approaches to understanding and assessing the impacts of drug checking services focus on behaviour change. There are several potential avenues for extending this work on drug taking behaviours including not only longitudinal methods (i.e. follow-up) but integration of theory. There are multiple contemporary behavioural change theories which could be applied to the setting of a harm reduction service. Theories such as the social cognitive learning theory [35] can be useful in understanding individual change, while broader approaches like the behaviour change wheel [36] can highlight service and system-level facilitators and barriers.

One of the most significant findings of the evaluation is that there was a dramatic increase in the proportion of respondents who reported that they would, as a result of the service, be more likely to access a health service to seek information about their drug use. Conversely, patrons reported that they would rely less on dealers, friends and online sources for information about drug-related harm and content. Drug checking patrons are typically considered to be difficult-to-reach, not-yet-problematic, but nevertheless a high-risk group of recreational drug users [37]. Thus, the impact of engaging these patrons with harm reduction support and information, which they may otherwise consider to be less trustworthy, is an important impact. This is particularly the case as Australia is starting to build a more integrated and robust network of Drug Early Warning Systems [38]. Ideally, these Early Warning Systems would be linked to drug checking services in the community, providing harm reduction information the broader community, not just those accessing a drug checking service.

### Limitations

As a small pilot study in a real world, there are limitations with the data set. In addition to missing questionnaire data (particularly the post-testing survey), the follow-up data set is small. Studies such as this, that use self-report data, particularly regarding illegal behaviour like drug use, are prone to social desirability bias; in this case, the under-reporting of socially disapproved behaviour. We do not believe that this is significant in this evaluation as (1) the participants were anonymous, and (2) the service operated on a non-judgemental basis, neither condoning nor opposing drug use.

The evaluation design did not include a representative sample of all the GTM festival attendees, nor of people interested in using drugs at the festival. Rather, its focus was on all the festival attendees who presented drugs for checking. Furthermore, while the current study did not include a control group, our design was adequate for examining the impact of pill testing and peer-delivered information on patrons’ attitudinal and knowledge outcomes at music festivals. Future research may consider the adoption of an experimental or quasi-experimental design where patrons may be randomly/non-randomly assigned to services with different specifications in order to answer research questions concerning the impact of pill testing with or without a peer-delivered information component, or various forms of testing (i.e. reagent, onsite or fixed-site pill testing).

This is the first independent evaluation in Australia to collect follow-up data and the contextual insight provided by the qualitative interview data brings value to the study, even if the number of respondents is small.

## Conclusions

This model of evaluation has provided insight into the process and outcomes of a trial drug checking service in the festival setting. In terms of feasibility, the questionnaire delivery and content provided the anticipated outcomes and we have shown that follow-up of service patrons is possible, although difficult. Strategies for engaging more patrons in follow-up data collection are needed.

Exploring the integration of drug checking service data with other data sources would provide useful analytical and practical outcomes, in particular interpreting the service utility in the wider context of drug markets, drug trends and health issues.

## Data Availability

The datasets generated and analysed during the current study are not publicly available due to requirement of the ANU Human Research Ethics Committee.
